# Supplementary material for: On the Origin of the Non‐Arrhenius Na‐ion Conductivity in Na3OBr
Source: Angew Chem Weinheim Bergstr Ger. 2023 Nov 14;135(51):e202314444. doi: 10.1002/ange.202314444 (PMC10952686; doi:10.1002/ange.202314444)
Supplement: Supplementary file 1 — Supporting Information [file ANGE-135-0-s001.pdf]

Angewandte Chemie
Eine Zeitschrift der Gesellschaft Deutscher Chemiker

Supporting Information

On the Origin of the Non-Arrhenius Na-ion Conductivity in Na_3OBr

*B. Darminto, G. J. Rees, J. Cattermull, K. Hashi, M. Diaz-Lopez, N. Kuwata, S. J. Turrell, E. Milan, Y. Chart, C. Di Mino, H. Jeong Lee, A. L. Goodwin, M. Pasta**

Synthesis of Na_3OBr

Na_3OBr was synthesised from sodium oxide (Sigma Aldrich, 80% Na_2O and 20% Na_2O_2) and sodium bromide (Sigma Aldrich, 99% NaBr) via a two-step solid-state reaction. Due to the hygroscopic nature of the precursors and the final product, all synthesis, material handling, and measurements were carried out in gloveboxes filled with argon (MBraun, $O_2 < 1$ ppm, $H_2O < 1$ ppm). Prior to the synthesis, both reagents used were dried at 120 °C for 12 h in a glass vacuum oven (Buchi). Due to the slow reaction kinetics between solids, ball milling was performed to achieve intimate mixing of the precursors and reduce the length scales for atomic interdiffusion and reaction. Sodium oxide and sodium bromide were loaded into a 45 ml ZrO_2 milling jar containing ten 5 mm diameter and five 10 mm diameter ZrO_2 balls. A planetary ball mill (Fritsch Puverisette 7 Premium) was used at 350 rpm for a total effective milling time of 10 hours with a 5 minute pause for every 10 minutes of milling. The ball-milled powder was then heated in a 15 ml cylindrical alumina crucible at 450 °C for 24 hours and left to cool down to room temperature inside the muffle furnace. To form the sample for ionic conductivity measurement, Na_3OBr was ground to powder and pressed into a 5 mm diameter cylindrical polyether ether ketone (PEEK) cell at 370 MPa for 3 minutes. The final thickness of the pellet was 300 µm.

Differential Scanning Calorimetry (DSC)

The DSC instrument was operated in air, so the sample was sealed in an aluminium hermetic pan inside the glovebox prior to the measurement to protect it from moisture. Measurements were performed between 25 and 300 °C with a heating rate of 5 °C min^{-1}.

Electrochemical Impedance Spectroscopy (EIS)

The EIS characterisation was carried out in a custom-built PEEK solid-state cell using gold powder (Alfa Aesar, gold powder, spherical, APS 0.5 – 0.8 µm) as the blocking electrodes.[1] Gold powder was added to one side of the cold-pressed pellet and then pressed at 370 MPa for 3 minutes before turning the pellet over to perform the same procedure on the opposite side. Using a screw on top of the cell, a uniaxial pressure of around 70 MPa was set by a torque wrench to ensure good contact. This pressure was calibrated using a load cell. The cell was then heated inside a muffle furnace in a glovebox for an hour at each data point before a measurement was performed using a two-probe configuration to a BioLogic MTZ35 frequency response analyser. Potentiostatic EIS (PEIS) measurements were conducted in the frequency range of 35 MHz to 0.1 Hz with a voltage amplitude of 10 mV. ZView software was used to simulate Nyquist plots based on the equivalent circuit depicted in Fig S1.

Ex-situ Scanning Electron Microscopy (SEM)

SEM measurements were conducted using a Thermo-Fisher Helios G4-CXe Plasma FIB (PFIB) instrument with energy-dispersive X-ray spectroscopy (EDX) functionality (Oxford Instruments). Both cold-pressed (CP) and hot-pressed (HP) pellets were mounted on a transportable SEM stage with carbon tape inside an Ar-filled glovebox before sealing in a Gatan iLoad sample transfer vessel. The stage was transferred from the vessel to the SEM chamber via a load-lock, avoiding exposure of the sample to air.

X-ray Diffraction (XRD)

Ex-situ X-ray powder diffraction measurements were performed on the as-synthesised Na_3OBr powder using a Rigaku Smartlab diffractometer (Cu Kα) to assess its phase purity. Synchrotron XRD measurements were performed on the I11 beamline of the Diamond Light Source operating with an X-ray wavelength of 0.825 318(3) Å. Diffraction patterns were collected in capillary transmission geometry using the Mythen2 Position Sensitive Detector, two data collections of 5 seconds each were taken at angles 0.25 degrees apart, then summed to account for gaps in the detector coverage. Over the temperature range of 25 to 300 °C, patterns were collected at approximately 2.5 °C intervals with a continuous heating rate of 6 °Cmin^{-1} using an FMB Oxford cyberstar hot air blower. All Rietveld refinements were carried out using the TOPAS-Academic software.[2] In total, 20 parameters were refined: 11 polynomial function parameters to fit the background, the lattice parameter, b_{eq} Na, b_{eq} Br, b_{eq} O, the scale parameter, and the 4 peak shape function parameters (pku, pkv, pkw, and pky).

Total scattering data were collected at I15-1 beamline at Diamond Light Source operating with an X-ray wavelength of 0.161 669 Å. Samples were put in 1mm borosilicate glass capillaries. Data for both background containers and samples were collected in a Q range from 0.3 to 25 A^{-1} and in a temperature range from 150 to 300 °C in 5 °C intervals. GudrunX routines were employed to subtract empty container, compton scattering and absorption from the data, and to normalise them via Krogh-Moe & Norman normalisation.[3–5]

Nuclear Magnetic Resonance (NMR) Spectroscopy

NMR measurements were performed at room temperature and at variable temperatures. All the room temperature ^{23}Na static solid-state NMR measurements were completed at magnetic field strengths of 20 T (ν_0 ^{23}Na = 264.6 MHz) and 23.5 T (ν_0 ^{23}Na = 224.9 MHz) using Bruker Neo consoles and 3.2 mm probes. The spectra were referenced to NaCl at 7.2 ppm,[6] and were recorded using a Hahnecho ($\pi/2$ - τ - π - τ) sequence with 400 kHz WURST (wideband, uniform rate, smooth truncation) excitation and refocussing pulses for broadband excitation of the ≈ 220 kHz wide resonances (at 20 T).[7,8] The variable temperature NMR measurements were made at National Institute for Materials Science (NIMS) using an ECA-500 (JEOL, Japan) spectrometer and a homemade probe.[9] The sample was packed into a quartz NMR tube SP-405 (SHIGEMI, Japan) and sealed in an Ar-filled globe box. The resonance frequency of ^{23}Na was 132.32 MHz. The chemical shift was referenced to the 1.0 M NaCl solution at 0 ppm. The temperature was controlled by a nitrogen gas flow.

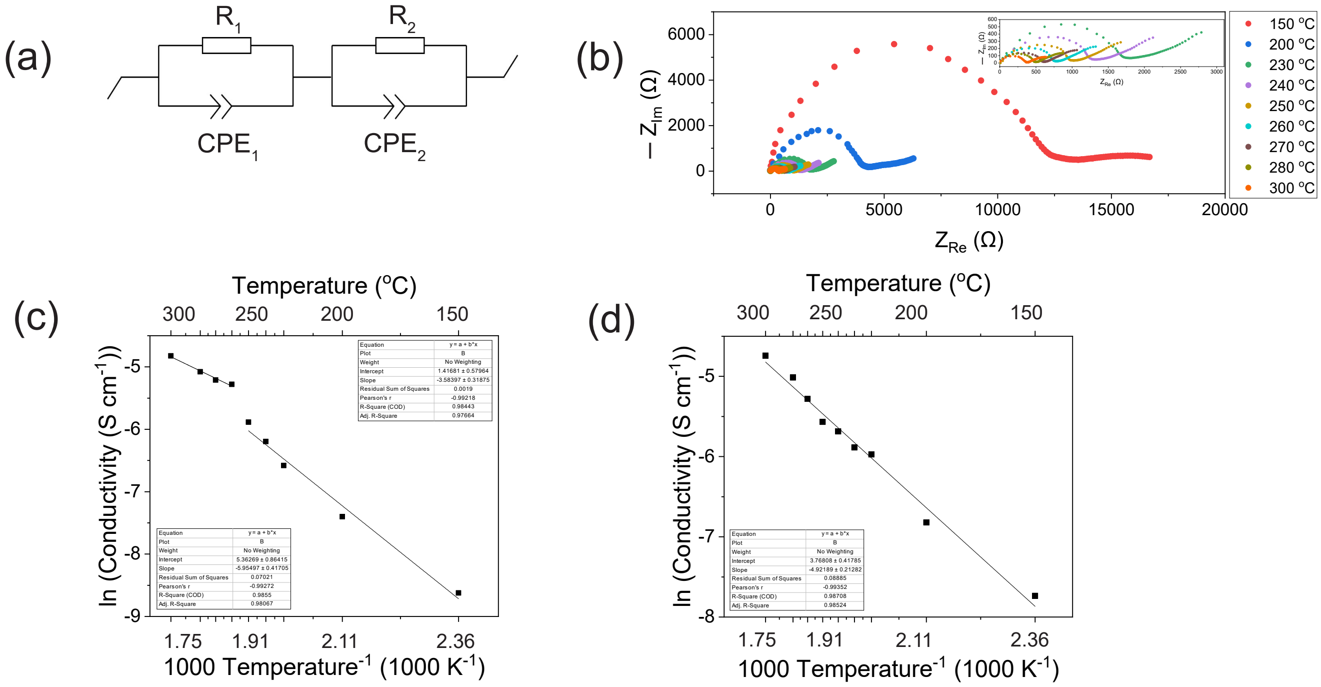

Figure S1. (a) Equivalent circuit used to simulate our experimental set-up. R_1 is the bulk resistance, CPE_1 is the bulk capacitance, R_2 is boundary resistance, and CPE_2 is boundary capacitance. (b) Nyquist plots of Na_3OBr at multiple temperatures between 25 °C and 300 °C. Zoomed in plots from 230 °C to 300 °C can be found in the inset. (c) Arrhenius plot of boundary ionic conductivity of Na_3OBr. (d) Arrhenius plot of bulk ionic conductivity of Na_3OBr with a step increase observed at 250 °C. Details of the linear plots can be found in the insets.

Table 1. Sequential Rietveld refinement results for Na_3OBr.

Temperature [°C]	R_{wp} [%]	Lattice Parameter [Å]	Lattice Parameter Error [Å]	$b_{eq\mathrm{Na}}$ [a.u.]	$b_{eq\mathrm{Na}}$ Error [a.u.]	$b_{eq\mathrm{Br}}$ [a.u.]	$b_{eq\mathrm{Br}}$ Error [a.u.]
25.5	1.022180	4.561558	0.000021	1.641498	0.054317	1.174098	0.040107
29.5	1.070940	4.562104	0.000022	1.682288	0.057362	1.187506	0.042266
30.2	1.050340	4.562022	0.000022	1.662280	0.056135	1.150041	0.041136
33.8	1.098070	4.562637	0.000023	1.694135	0.059128	1.190575	0.043534
35.8	1.114310	4.562716	0.000023	1.716716	0.060192	1.184791	0.044092
38.6	1.120320	4.563202	0.000023	1.732066	0.060764	1.221527	0.044771
38.9	1.108310	4.563253	0.000023	1.737458	0.060102	1.220345	0.044256

41.8	1.092120	4.563737	0.000023	1.750327	0.059553	1.224984	0.043878
43.4	1.067490	4.564008	0.000022	1.750455	0.058234	1.256517	0.043170
44.6	1.063110	4.563875	0.000022	1.781834	0.058125	1.216448	0.042544
47.8	1.028520	4.564485	0.000022	1.753494	0.056319	1.275530	0.041925
51.7	1.013890	4.565123	0.000021	1.795308	0.055785	1.268658	0.041247
51.9	1.016600	4.564780	0.000021	1.796136	0.055968	1.266525	0.041358
52.3	1.013310	4.564857	0.000021	1.812125	0.055961	1.298888	0.041531
58.3	1.001380	4.565673	0.000021	1.817225	0.055458	1.304760	0.041206
58.2	1.020540	4.565856	0.000022	1.839877	0.056807	1.306292	0.042068
60.2	1.023250	4.566016	0.000022	1.839279	0.056869	1.302573	0.042089
61.4	1.013820	4.566159	0.000021	1.862333	0.056659	1.335295	0.042089
64.4	1.017710	4.566436	0.000022	1.862421	0.056895	1.320784	0.042138
67.2	1.029210	4.566869	0.000022	1.907565	0.057962	1.364860	0.043025
69.8	1.050380	4.567208	0.000022	1.918637	0.059330	1.356367	0.043879
72.3	1.061780	4.567557	0.000023	1.982388	0.060402	1.381109	0.044507
74.7	1.083880	4.567831	0.000023	1.959962	0.061667	1.381132	0.045589
76.4	1.077080	4.568076	0.000023	2.018258	0.061699	1.405451	0.045495
78.0	1.063500	4.568285	0.000023	1.987839	0.060722	1.393819	0.044868
79.7	1.087430	4.568511	0.000023	1.991809	0.062223	1.410599	0.046126
81.4	1.070960	4.568735	0.000023	1.996786	0.061360	1.448284	0.045798
84.2	1.060980	4.569042	0.000023	1.985291	0.060724	1.404863	0.045033
86.8	1.030940	4.569382	0.000022	1.988088	0.059100	1.420591	0.043951
88.3	1.033140	4.569568	0.000022	2.012918	0.059441	1.431820	0.044175
90.8	0.989550	4.569885	0.000021	2.034860	0.057131	1.408760	0.042159
92.6	0.988320	4.570150	0.000021	2.001152	0.057016	1.463944	0.042779
94.4	0.998130	4.570372	0.000021	2.028245	0.057737	1.460003	0.043115
96.6	0.982790	4.570600	0.000021	2.048450	0.056889	1.479915	0.042517
98.3	0.988120	4.570851	0.000021	2.077601	0.057530	1.465690	0.042737
100.4	0.991480	4.571093	0.000021	2.073464	0.057718	1.468326	0.042911
103.1	1.032090	4.571414	0.000022	2.103607	0.060423	1.466802	0.044762
104.4	1.057630	4.571630	0.000023	2.156624	0.062278	1.496599	0.046057
106.8	1.061870	4.571963	0.000023	2.152221	0.062637	1.521886	0.046598
109.3	1.080680	4.572189	0.000023	2.159350	0.063845	1.506895	0.047305
110.9	1.077580	4.572454	0.000023	2.185579	0.063903	1.553296	0.047619
112.5	1.064620	4.572691	0.000023	2.210308	0.063456	1.570515	0.047317
115.3	1.041060	4.572978	0.000023	2.201779	0.061831	1.559007	0.046004
116.6	1.031270	4.573211	0.000022	2.219332	0.061472	1.620377	0.046207
118.6	1.007200	4.573457	0.000022	2.222875	0.060028	1.579716	0.044733
121.0	0.988990	4.573753	0.000021	2.252637	0.059204	1.641707	0.044492
122.9	0.990890	4.573971	0.000021	2.273752	0.059444	1.629130	0.044442
125.0	0.987780	4.574221	0.000021	2.254900	0.059154	1.608957	0.044163
126.8	0.993050	4.574459	0.000021	2.296747	0.059633	1.641235	0.044565
128.9	1.027150	4.574727	0.000022	2.297713	0.061918	1.665525	0.046532
131.1	1.015570	4.574989	0.000022	2.298631	0.061334	1.640609	0.045871
133.1	1.034150	4.575227	0.000022	2.329541	0.062585	1.671565	0.046918
135.2	1.022070	4.575517	0.000022	2.308294	0.061683	1.642435	0.046091
137.2	1.023730	4.575796	0.000022	2.317716	0.061950	1.676293	0.046595
139.2	1.036420	4.576055	0.000022	2.360809	0.062999	1.672897	0.047104
141.2	1.034220	4.576293	0.000022	2.361023	0.062923	1.696987	0.047271
143.1	1.022480	4.576563	0.000022	2.360733	0.062183	1.728142	0.047037
145.1	1.011270	4.576754	0.000022	2.341543	0.061352	1.721708	0.046439
146.9	1.009480	4.577040	0.000022	2.367194	0.061458	1.748641	0.046629
148.4	1.006580	4.577217	0.000022	2.370490	0.061171	1.715567	0.046076
151.8	1.003750	4.577588	0.000021	2.448178	0.061545	1.761531	0.046329
154.0	0.993770	4.577950	0.000021	2.398722	0.060709	1.785453	0.046180
154.5	0.991880	4.578001	0.000021	2.434332	0.060710	1.761441	0.045762
157.3	0.985480	4.578329	0.000021	2.478667	0.060582	1.812169	0.045855
159.6	0.986930	4.578645	0.000021	2.482958	0.060621	1.794336	0.045616
161.4	1.007770	4.578885	0.000021	2.510194	0.061953	1.795025	0.046451

163.4	1.021090	4.579125	0.000022	2.473405	0.062471	1.837628	0.047459
165.2	1.013610	4.579364	0.000021	2.539701	0.062461	1.819217	0.046861
167.3	1.039610	4.579648	0.000022	2.552492	0.064046	1.878824	0.048528
169.5	1.054850	4.579905	0.000022	2.562635	0.064998	1.880689	0.049135
171.5	1.050240	4.580157	0.000022	2.571787	0.064761	1.864617	0.048773
173.7	1.057460	4.580451	0.000022	2.584111	0.065234	1.875047	0.049143
175.7	1.042650	4.580703	0.000022	2.556035	0.064116	1.869714	0.048404
178.1	1.025750	4.580968	0.000021	2.610686	0.063316	1.885098	0.047598
179.4	1.017140	4.581205	0.000021	2.609457	0.062621	1.873848	0.046968
181.7	0.985880	4.581470	0.000020	2.613781	0.060620	1.913848	0.045783
183.7	0.995620	4.581754	0.000020	2.620119	0.061023	1.909545	0.045964
186.0	0.973780	4.581984	0.000019	2.615288	0.059686	1.911499	0.045012
188.1	0.968700	4.582286	0.000019	2.658527	0.059461	1.927788	0.044740
190.2	0.982530	4.582515	0.000019	2.588594	0.059801	1.937567	0.045424
192.2	0.985210	4.582799	0.000019	2.646039	0.060269	1.985564	0.045880
193.8	1.013680	4.582987	0.000020	2.619018	0.061787	1.965825	0.047041
196.1	1.014770	4.583288	0.000020	2.603069	0.061788	1.963376	0.047094
198.1	1.040380	4.583514	0.000020	2.693667	0.063931	2.029609	0.048776
200.1	1.038740	4.583796	0.000020	2.673244	0.063567	1.999470	0.048299
202.0	1.056670	4.584052	0.000020	2.685271	0.064611	1.989251	0.048898
204.2	1.061900	4.584290	0.000020	2.686281	0.064846	2.002083	0.049183
206.1	1.083280	4.584578	0.000021	2.740929	0.066519	2.040210	0.050439
208.2	1.090290	4.584827	0.000021	2.735744	0.066869	2.059365	0.050909
210.3	1.084280	4.585079	0.000020	2.702444	0.066143	2.057508	0.050513
212.3	1.069260	4.585349	0.000020	2.744731	0.065509	2.092230	0.050101
214.2	1.060950	4.585572	0.000020	2.743554	0.065141	2.089594	0.049796
216.0	1.045690	4.585801	0.000019	2.794755	0.064405	2.096600	0.048976
218.5	1.034440	4.586084	0.000019	2.756536	0.063582	2.138155	0.048982
220.5	1.041130	4.586398	0.000019	2.806801	0.064248	2.098070	0.048809
222.5	1.037340	4.586572	0.000019	2.789912	0.063800	2.083386	0.048403
224.6	1.040320	4.586841	0.000019	2.780892	0.063946	2.103771	0.048772
226.3	1.061310	4.587078	0.000019	2.806082	0.065435	2.141556	0.050117
228.3	1.091800	4.587373	0.000020	2.846150	0.067408	2.120600	0.051161
230.5	1.099650	4.587600	0.000020	2.815234	0.067693	2.119831	0.051526
232.5	1.141310	4.587902	0.000021	2.897480	0.070990	2.155864	0.053885
234.9	1.147290	4.588162	0.000021	2.875962	0.071099	2.150777	0.053998
236.8	1.176220	4.588433	0.000021	2.873062	0.072970	2.155952	0.055505
238.6	1.197310	4.588650	0.000021	2.903616	0.074477	2.151803	0.056377
240.6	1.206460	4.588896	0.000022	2.882066	0.075014	2.171504	0.057174
242.6	1.221840	4.589165	0.000022	2.894738	0.075894	2.166150	0.057696
244.4	1.222980	4.589366	0.000022	2.900281	0.076217	2.275002	0.059139
245.8	1.230040	4.589569	0.000022	2.876689	0.076683	2.261530	0.059513
248.6	1.230160	4.589858	0.000022	2.933898	0.077337	2.277839	0.059834
250.5	1.200720	4.590165	0.000021	2.912927	0.075510	2.352056	0.059328
252.8	1.156960	4.590476	0.000020	2.841799	0.072095	2.345049	0.056969
254.9	1.135160	4.590752	0.000020	2.821947	0.070565	2.328832	0.055701
256.9	1.098570	4.590995	0.000019	2.888071	0.069072	2.344901	0.054278
259.0	1.082420	4.591258	0.000019	3.011485	0.069507	2.350454	0.053834
261.1	1.117160	4.591526	0.000020	3.009343	0.072207	2.293061	0.055358
263.2	1.166300	4.591792	0.000021	3.046364	0.075439	2.293431	0.057570
265.3	1.211210	4.592066	0.000022	3.026213	0.078456	2.321632	0.060367
267.3	1.255630	4.592346	0.000023	3.091264	0.082218	2.351029	0.063174
269.1	1.304030	4.592608	0.000024	3.105611	0.085627	2.364193	0.065871
271.4	1.388870	4.592924	0.000025	3.058499	0.090824	2.401381	0.070793
273.3	1.386770	4.593128	0.000025	3.045875	0.090579	2.424539	0.071043
275.2	1.405770	4.593409	0.000026	3.058407	0.092279	2.363007	0.071475
277.1	1.398620	4.593680	0.000026	3.107752	0.092495	2.398858	0.071712
279.2	1.389660	4.593939	0.000026	3.082468	0.091760	2.390384	0.071265
281.3	1.369710	4.594217	0.000025	3.063352	0.090504	2.429325	0.071050

283.2	1.315440	4.594514	0.000024	3.150048	0.087579	2.478143	0.068665
285.2	1.304750	4.594806	0.000024	3.072160	0.086283	2.480365	0.068394
287.3	1.247850	4.595054	0.000022	3.032978	0.082044	2.487612	0.065414
289.3	1.211410	4.595335	0.000022	3.041706	0.079564	2.538582	0.064003
291.4	1.155430	4.595616	0.000021	3.094840	0.076691	2.595872	0.062017
293.4	1.129980	4.595879	0.000020	3.021956	0.074948	2.559139	0.060726
295.4	1.119240	4.596141	0.000020	3.010138	0.074370	2.549830	0.060205
297.4	1.139960	4.596422	0.000021	3.063871	0.076676	2.537890	0.061542
299.3	1.176820	4.596691	0.000022	3.091901	0.079723	2.486193	0.063171
300.4	1.196080	4.596833	0.000022	3.159427	0.081841	2.488018	0.064368

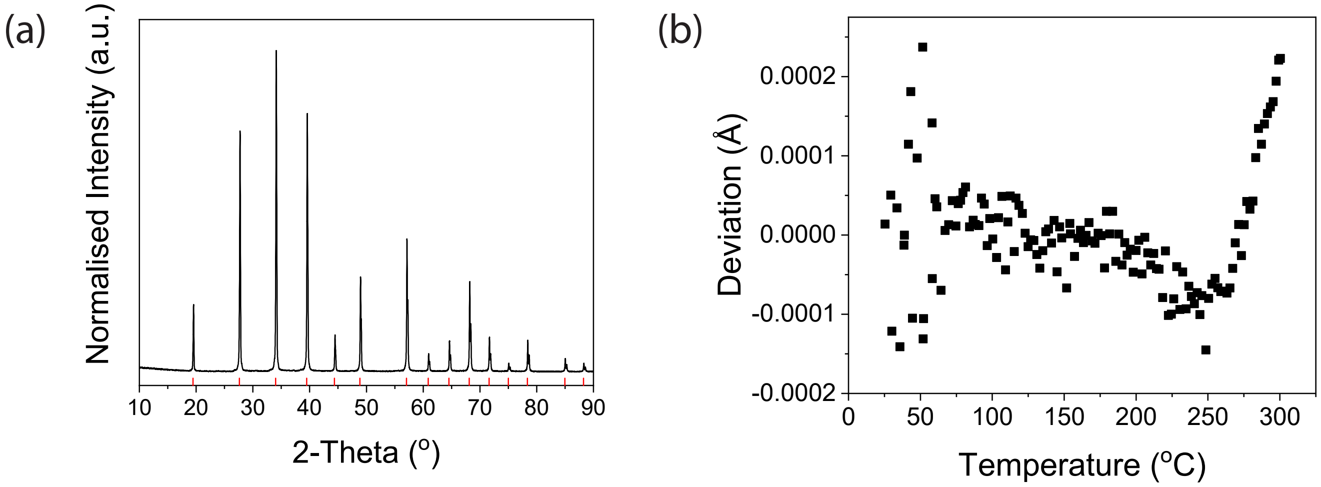

Figure S2. (a) Ex-situ X-ray diffractogram of Na_3OBr. (b) Deviation of the lattice parameter of Na_3OBr from a linear trendline as a function of temperature.

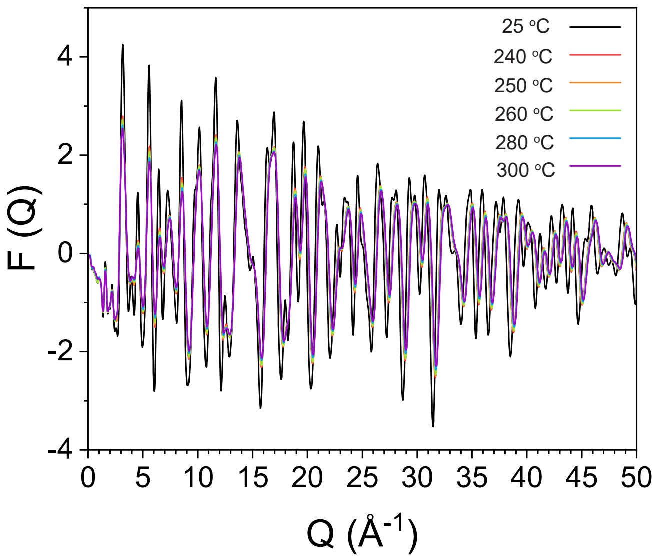

Figure S3. X-ray total structure factor *F(Q)* of Na_3OBr.

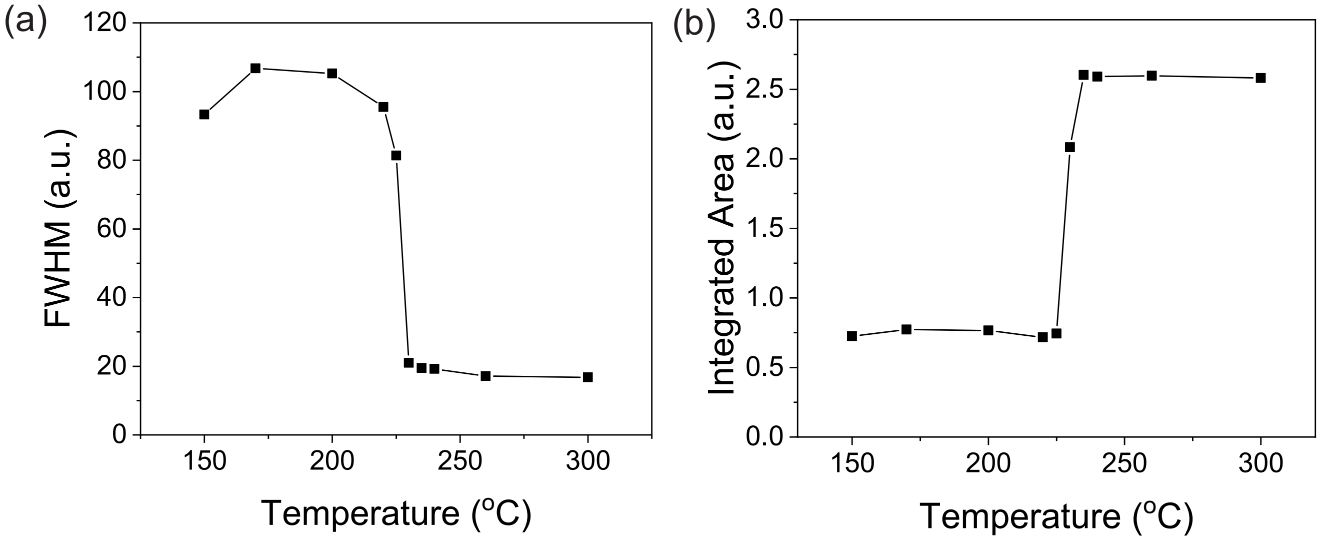

Figure S4. (a) Full-width half maximum and (b) integrated area under the impurities peak around 0 ppm as a function of temperature.

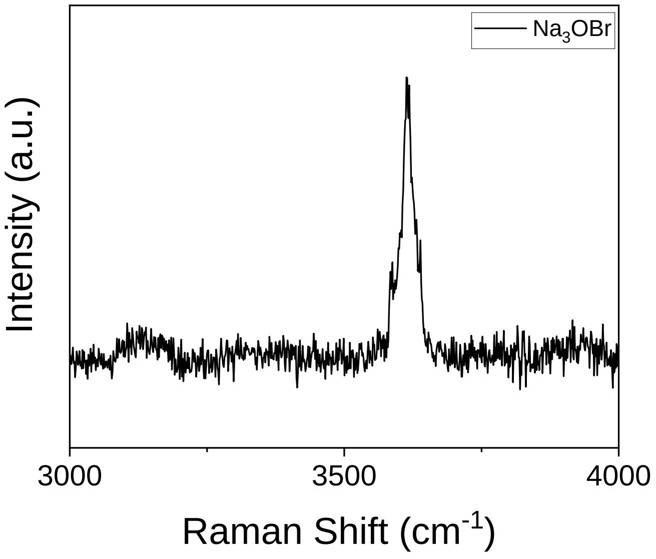

Figure S5. Raman spectra from $3000\,\mathrm{cm}^{-1}$ to $4000\,\mathrm{cm}^{-1}$ showing a peak at $3620\,\mathrm{cm}^{-1}$, which belongs to NaOH.

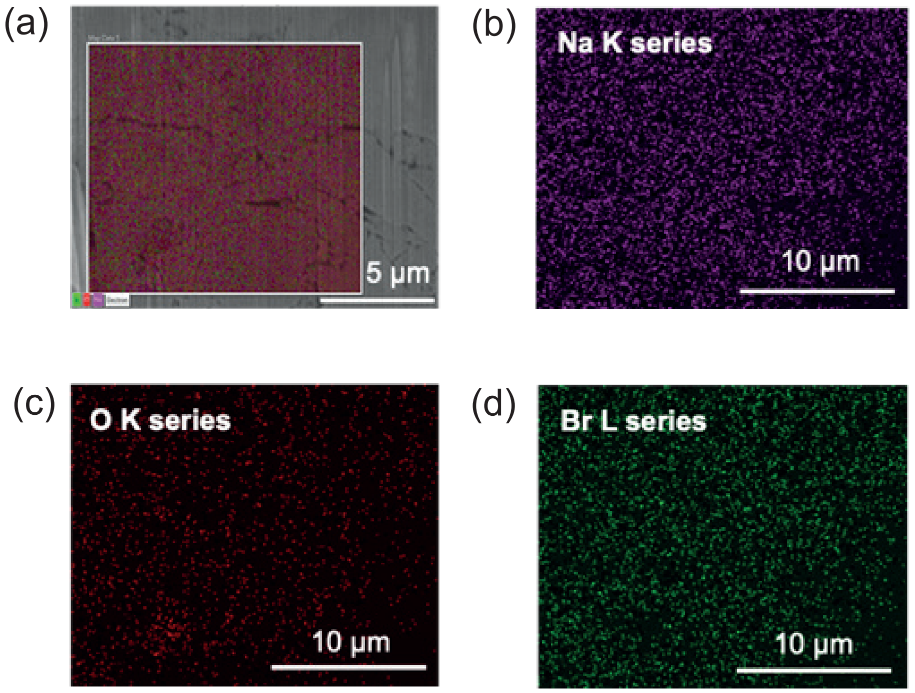

Figure S6. (a) Cross-sectional secondary electron image of the cold-pressed Na_3OBr pellet before variable temperature EIS measurements. Corresponding EDX analysis of the different elements: (b) Na (purple), (c) O (red), and (d) Br (green). These images illustrate that Na, Br, and O are homogeneously distributed throughout the pellet.

References

[1] C. Doerrer, I. Capone, S. Narayanan, J. Liu, C. R. Grovenor, M. Pasta, P. S. Grant, *ACS Appl. Mater. Interfaces* **2021**, *13*, 37809–37815.

[2] A. A. Coelho, Topas Academic V6 **2016**.

[3] J. Krogh-Moe, *Acta Cryst.* **1956**, *9*, 951–953.

[4] N. Norman, *Acta Cryst.* **1957**, *10*, 370–373.

[5] A. K. Soper, GudrunN and GudrunX: programs for correcting raw neutron and X-ray diffraction data to differential scattering cross section, Technical report **2011**.

[6] K. J. MacKenzie, M. E. Smith, Multinuclear Solid-State NMR of Inorganic Materials, in *Pergamon Materials Series*, volume 6, Pergamon **2002**.

[7] E. Kupce, R. Freeman, *J. Magn. Reson., Ser. A* **1995**, *117*, 246–256.

[8] E. Kupce, R. Freeman, *J. Magn. Reson., Ser. A* **1995**, *115*, 273–276.

[9] K. Hashi, S. Ohki, Y. Mogami, A. Goto, T. Shimizu, *Anal Sci.* **2021**, *37*, 1477–1479.
